# Artificial Reef Effect in relation to Offshore Renewable Energy Conversion: State of the Art

**DOI:** 10.1100/2012/386713

**Published:** 2012-12-23

**Authors:** Olivia Langhamer

**Affiliations:** Department of Biology, Norwegian University of Science and Technology, Høgskoleringen 5, 7491 Trondheim, Norway

## Abstract

The rapid worldwide growth of offshore renewable energy production will provide marine organisms with new hard substrate for colonization, thus acting as artificial reefs. The artificial reef effect is important when constructing, for example, scour protections since it can generate an enhanced habitat. Specifically, artificial structures can create increased heterogeneity in the area important for species diversity and density. Offshore energy installations also have the positive side effect as they are a sanctuary area for trawled organisms. Higher survival of fish and bigger fish is an expected outcome that can contribute to a spillover to outer areas. One negative side effect is that invasive species can find new habitats in artificial reefs and thus influence the native habitats and their associated environment negatively. Different scour protections in offshore wind farms can create new habitats compensating for habitat loss by offshore energy installations. These created habitats differ from the lost habitat in species composition substantially. A positive reef effect is dependent on the nature and the location of the reef and the characteristics of the native populations. An increase in surface area of scour protections by using specially designed material can also support the reef effect and its productivity.

## 1. Introduction

A changing climate due to fossil fuel emissions and increasing energy demands leads to a new focus on renewable energy sources. Therefore, diverse countries in Western Europe have plans to build and commercialise offshore renewable energy resources along their coastlines and this industry is going to grow significantly. Offshore wind, for example, has a much higher potential to produce energy than terrestrial farms and may contribute with about 140 GW in the year 2030 and would thus produce more than 10% of Europe's electricity [1]. Moreover, offshore wave power is developing fast, gaining increased attention [2], and with the first units in operation [3, 4]. All in all, offshore renewable energy production is a fast growing industry and typically spread out over large areas on the seafloor. Cables, concrete or steel piles, and foundations will be the typical hard bottom structures in an offshore renewable energy park. Still, there is a gap of knowledge in general and local effects of large scale offshore development on these already stressed marine environments. Today's research on offshore energy conversion is focused on fish [5], marine mammals [6], effects of electromagnetic fields [7], noise [8], hydrodynamic changes, and benthic communities [9, 10]. Concerning sea cables, impact studies showed that there were no general negative impacts on species abundance, composition and biomass of infauna, and species specific reactions in some elasmobranch fish [11–13].

Still, research on offshore renewable industry is in the beginning phase. Both long-term studies and large scale effects are topics of high scientific value that need to be prioritised. Furthermore, there is a lack of both replication and baseline studies that are very essential for reliable results that can be used for more general decision makings. So far, the impacts during the construction phase of offshore installations seem to be at its highest during this phase, including a lot of noise, boat traffic, cable laying, and seabed disruptions. During maintenance, the noise generation of the turbines/generators, vibrations from the installations, and their physical presence may be some of the critical impact factors [4, 7]. The marine environment may on the other hand benefit from the installation of offshore renewable energy, since trawling will be excluded and new hard substrate will be introduced. In this paper I will discuss the opportunities of offshore renewable energy as a habitat enhancement. Specifically for threatened or commercially interesting species, such as for example, juvenile whiting, cod and lobsters this may lead to a great benefit for nature conservation. There is a high plausibility that offshore energy installations act as artificial reefs [5, 14–16] which in its way can support both environmental and commercial interests. 

## 2. Colonisation of Offshore Structures

Artificial offshore constructions will inevitably be colonized by a number of organisms. This should be considered when constructing for example scour protections with their potential to enhance the reef effect for higher biodiversity or commercial interesting species. Artificial reefs generally hold greater densities and biomass of fish and decapods, and provide higher catch rates, compared to surrounding soft bottom areas, and in several cases also in relation to adjacent natural reefs [17–24]. There are, however, some studies that show no significant impacts of artificial reefs on fish assemblages [25]. The proposed reasons for higher abundance and diversity of fish on and around artificial reefs differ among organisms. The most important seems to be the provision of shelter from both predation and water movements, and enhanced feeding grounds. Fish also seem to use the structures as reference points for spatial orientation [18, 19, 26].

Coming at the base of wind power farms, scouring protections may have a potential in terms of altering the nature of the seabed in the vicinity of wind farms. In that way different shapes and sizes may create different habitats and thus dictate what kind of organisms colonize for living and feeding. Wind farms are usually constructed on soft bottom substrate for technical reasons, and this contributes to higher complexity in three-dimensional scale. Therefore, scour protections have the potential to turn exposed, biodiversity-poor soft bottoms into species rich ecosystems. When the conditions are ideal, wind park foundations will become heavily colonized by organisms abundant in the water mass or nearby hard-bottom habitats. The colonisation is highly dependent on sufficient number of larvae and suitable environmental conditions [27]. On the other hand habitat mitigation can occur depending on the location of the renewable energy installations. Therefore, adequate location decision is important to prevent negative impacts in areas where red-listed or key-species exist. 

Recruitment of marine organisms primarily occurs in two different ways when new constructions such as scour protections are set in place: by migration from the surrounding substrate or by settling of larvae. The recruitment will be governed by the local hydrodynamic regime [28] carrying the larvae to the wind farm, and then it will depend on its material and textures [29], and on the location of the scour protection in respect of water depth [30], salinity and temperature [31–33], and so forth. An initial macromolecule film, bacteria, microalgae, and fungi colonising the surface of scour protections may either favour or deter the settlement of larvae [34]. The colonisation will often have a characteristic succession, starting with diatoms and filamentous algae, followed by barnacles, and thereafter by a more diverse community [35]. There will be differences in the composition of fouling communities at particular depths on the scour protections. However, there is a high probability that scour protections will create increased heterogeneity in the area that is of great importance for species diversity and density. The size, diversity, and density of organisms on and in an artificial reef are conditional on the number and size of niches and not necessarily the presence of food. The conditions for the supply of nutrients are well established since offshore energy installations in shallow waters (<30 m) are built in areas with higher water turbulence efficiently transporting food, oxygen, and carbon dioxide. The extent to which scour protections may attract marine organisms and the species attracted will largely be dictated by the design of the components of the installation, with structural complexity of exposed surfaces being an important factor [36, 37].

Structural complexity appears to be a condition for many productive and intricate environments such as coral reefs, mangroves, and sea grass meadows. These environments are productive, not only because they act as a substrate that have a great turnover, but also because they offer a high degree of substrate complexity and an extensive spectrum of niche sizes, which are advantageous for young and juvenile organisms. One topic that is usually discussed when establishing artificial reefs is whether the reefs actually produce new biomass, or if it only aggregates or redistributes biomass. This depends mainly on the species and its limitation by food, refuge, territory, and/or behavioural requirements [18, 38]. For other species and in other regions> with different environmental conditions, artificial reefs do only redistribute or aggregate biomass [18]. However, studies on the production/aggregation theory are lacking, mostly because they are relatively short term and scarce in appropriate control sites or replications [39].

## 3. No-Trawling Areas/Sanctuary Areas

The deployment of artificial reefs in regions where there are no rocky bottoms may be important for specific hard substrate species [40]. Bottom trawling and dredging on soft sediments has been conducted over most of the world's continental shelves for decades and includes heavy fishing gear that is towed over the seafloor. These types of fishing stir up bottom sediments and loading suspended solids into the water column and have an immense and chronic impact on the marine environment [41, 42]. As a result of these processes, vulnerable species will be reduced, as well as biodiversity, production, and biomass in general [43–45]. Furthermore, ecological changes towards more opportunistic organisms due to removal of biomass are expected [46], and possible domino effects on the ecological relationships and food webs may be caused by the selective removal of some benthic species [47]. Additionally, about 70% of the marine fish stocks are overexploited or depleted due to trawling [48]. The primary dispute over trawling concerns the magnitude and duration of these impacts. An earlier meta-analysis showed that the large-scale effects of trawling are complex and depend on many factors including habitat, benthic composition, trawl intensity, and trawl gear types [49].

Over the last few decades the needs of protected areas where all fisheries and other forms of extraction of organisms are excluded have become a major focus in marine ecology, fisheries management, and conservation biology [50, 51]. There has been a growing interest for establishing no-trawling zones (NTZ), since they have been proposed to be efficient and inexpensive ways of maintaining and managing fisheries, while simultaneously conserving biodiversity and meeting other conservation objectives as well as human needs [50, 51]. NTZs established around the world range in size from a fraction of 1 km^2^ to 10 km^2^ or more. Around 0.04% of the world's oceans are currently designated as NTZ, in which all fishing is banned and can enable the ecosystem within the area to recover (at least partially) from the effects of fishing. Thus, NTZs can be defined as Marine Protected Areas in which the extraction of living and nonliving resources is permanently prohibited, except that they are necessary for monitoring or research to evaluate effectiveness. 

Establishing offshore energy parks will make it impossible to trawl close by the devices since there is always in a certain safety zone preventing entanglement of fishing gear. Trawling will be prohibited or limited in these safety zones that cover some km^2^, and accordingly, these offshore parks may act as NTZs, mitigating habitat losses and degradation. In these areas juvenile fish will have a higher chance to survive, and even older, bigger fish will improve survival rates, and in this way contributing to a spillover effect. One risk might be higher fishing pressure outside those parks where no safety zone exists since there might be a higher productivity within offshore parks leading to a spill over effect.

## 4. Invasive/Nonindigenous Species

One mitigating effect of offshore renewable energy on the local biodiversity may occur due to colonization by invasive species. Ever since international shipping started, marine organisms have been distributed all over the world by ballast water or as fouling on boat hulls. This introduction of alien species has dramatic ecological effects, since it can be a threat to global biodiversity [52, 53] and lead to local extinctions and fishery collapses [53]. Artificial hard substrates offer habitats for a large number of invasive species normally attached to rocky reefs [54]. In general, artificial structures do not host exactly the same species as a natural hard substrate [55, 56]. The installation of offshore renewable energy parks may not only introduce hard substrata in otherwise sandy-dominated bottoms, but can also provide new habitats for invasive species. Different hydrodynamics, such as more shelter due to new structures may lead to colonization of organisms very different to those on nearby hard substrates and thereby establish and spread nonindigenous species [57]. On wind turbine constructions in the North Sea and in the Baltic Sea the presence of alien species has been recorded [58–60] and may provide stepping-stones for spread, which could facilitate the establishment of the new taxa in the recipient region. 

## 5. Scour Protections in Offshore Wind Farms

Scour or erosion is the removal of granular seabed material around coastal structures by hydrodynamic forces. Scour around wind turbines is a major industrial and engineering problem and can result in serious (up to 10 m deep) sediment reduction around the foundations [61]. So far, there are some common procedures to prevent scouring [62].

### 5.1. Synthetic Fronds

**Figure 1:**
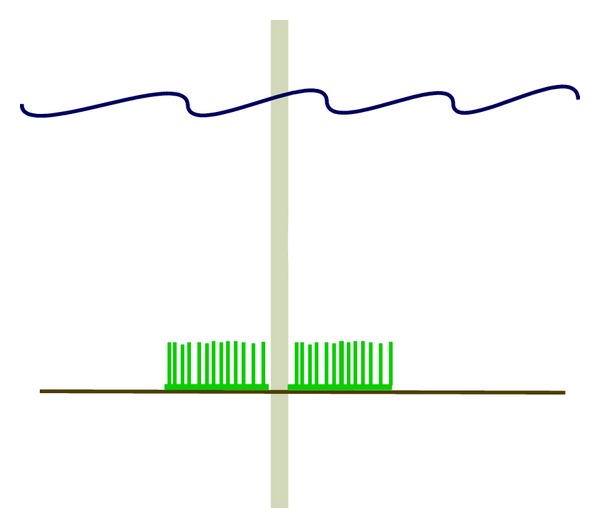
Illustration of polypropylene fronds designed as scour protection around monopile offshore wind turbines.

Polypropylene fronds that resemble seaweed beds can be used as scour control. They are fixed in concrete and anchors on the seafloor and form mattresses (Figure 1). The fronds slow down the local current and cause suspended particulate matter to settle. This matter accumulates over time to build up a sand or sediment bank, effectively reinstating the seabed. This fibre-reinforced bank will then resist further erosion. An installation in larger depths will make fewer problems with fouling organisms and debris. Around the wind power pilings the mats are building up a cohesive underwater sandbank and thus prevent scouring [63]. 

Synthetic fronds can mimic a seagrass environment, and thus may be used by several different species as habitat, feeding ground, shelter from predation, and nursery ground [64, 65]. A three-dimensional design of the new environment and sediment stabilization will support a rich and diverse invertebrate and fish fauna. Synthetic sea fronds may have a positive artificial reef effect since they create new habitat and thus increase the carrying capacity of an area and its ecological functioning. In contrast to sparse areas of the seabed (where offshore wind farms generally are placed), carrying capacity of more complex habitats will be higher [66]. Considering net habitat gain and losses the synthetic fronds will establish a reduced habitat availability because their surfaces give less space for colonization compared to the area they cover (Table 1) [16]. Still they will contribute to an increased ecological functioning since they create new habitat and thus heterogeneity.

### 5.2. Gravel/Boulder Protections

**Figure 2:**
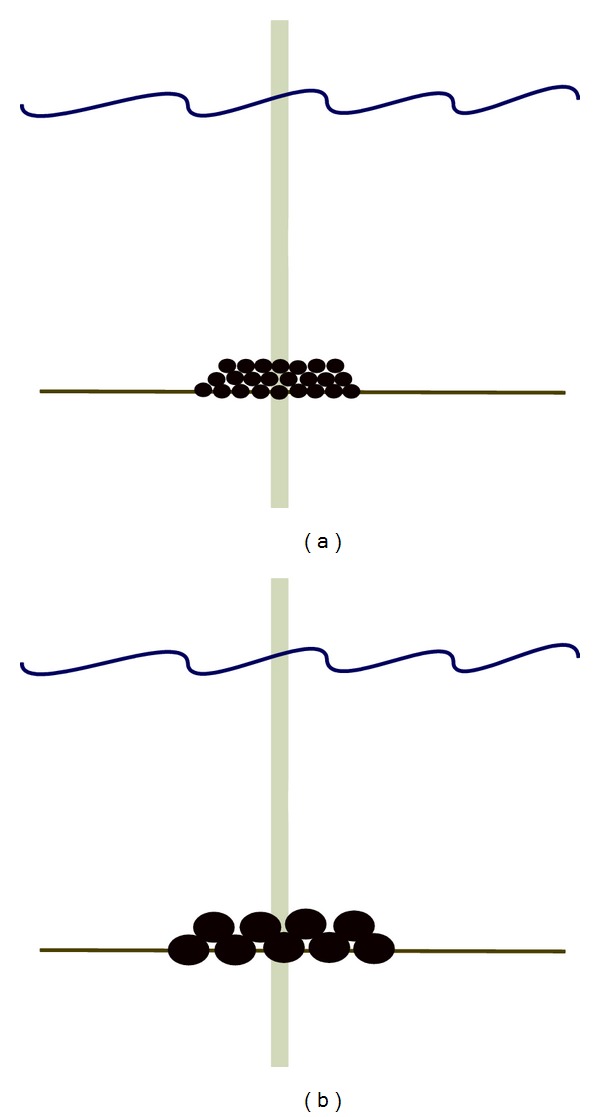
Illustrations of gravel (a) and boulder (b) protections around a monopile offshore wind power foundation.

Another inexpensive method of scour protection is to cover the base of wind turbines with bigger boulders and/or gravel (Figure 2). Here, one or more layers are aggregated around the turbine base to shape a circular reef of 10–15 m radius with the foundation at the centre. At the Danish Horns Rev this method is applied for protecting wind turbines. 

These kinds of scour protections are hard substrate environments that may be colonized by first barnacles, tube worms, and sea squirts. Later on, motile organisms such as lobsters, crabs as well as reef fish (wrasse, conger eel) can occur. Hard substrate generally have a higher biodiversity and species abundance than surrounding soft bottoms [67]. In comparison to boulders, gravel protections will result in low diversity and abundance of organisms due to a more unstable environment. Out of nature conservation perspectives that alternative is the least desired and low colonisation of hydrodynamically resistant species such as polychaetes, bivalves, echinoderms, and crustaceans (*Liocarcinus *spp., *Pagurus *spp.) is expected. In the case of low currents, hydroids, sea anemones, and bryozoans may occur. 

#### 5.2.1. Case Study: Danish Horns Rev

The established offshore wind farm covers approximately 14 500 m^2^ including scour protection. The whole offshore wind farm covers a total area of 27.5 km^2^. That gives an impact on the altered habitats in the range of 0.5% of the area of the offshore wind farm and the total loss of habitat would affect less than 0.1% of the bottom fauna within the site [68]. Colonisation processes at Horns Rev include fouling by algae and invertebrates [60]. Introduction of epifouling communities have increased the general biodiversity in the wind farm area and a succession in the benthic community and biodiversity has been observed. Evidence that the hard bottom substrates provide habitat as nursery grounds for larger and more mobile species has been shown for the edible crab *Cancer pagurus* [60].

A very high variation and differences in faunal assemblages on the scour protection between different sampling sites were shown. Still, some similarities between different zones mainly reflecting different type of substrate were demonstrated [60]. At the scour protection, sea anemones and the soft coral *Alcyonium digitatum *contributed with high percentages to the total biomass. Loss of infauna habitats has been replaced by hard bottom habitats providing an estimated 60 times increase in the availability of food for fish and other organisms in the wind farm area compared to the native infauna biomass.

Seasonal variations in fish fauna diversity were found in schools of cod often observed at the scour protections as well as individuals of benthic fish species [60]. Number of fish was higher in September than in March probably due to feeding on crustaceans that aggregated on scour protections.

Two designated species have been found in the Horns Rev area, the bristle worm *Sabellaria *presumably the ross worm *S. spinulosa *and the white weed *Sertularia cupressina*, which both are regarded as threatened or red listed in the Wadden Sea area [60].

Looking at benthic communities, the Danish monitoring studies observed that the introduction of the turbine foundations and scour protections onto seabed that previously consisted of relatively uniform sand have increased habitat heterogeneity [60]. Local changes to benthic communities have occurred, affecting typical fauna communities, with most aquatic animals living in the seabed, to hard bottom communities with increased abundance and biomass. There was a massive colonisation by common mussels, and a cover of algae was found shifting from an initial colonisation of filamentous green algae to a more diverse and permanent vegetation of green, brown, and red algae (Leonard and Pedersen, 2005). The Horns Reef wind farm will act as no-take zone since a cable protection zone of 200 m is going to be established around the wind farm and the cable where anchoring and fishing will not be allowed. 

### 5.3. Alternative Designs to Enhance Biomass and Diversity

**Figure 3:**
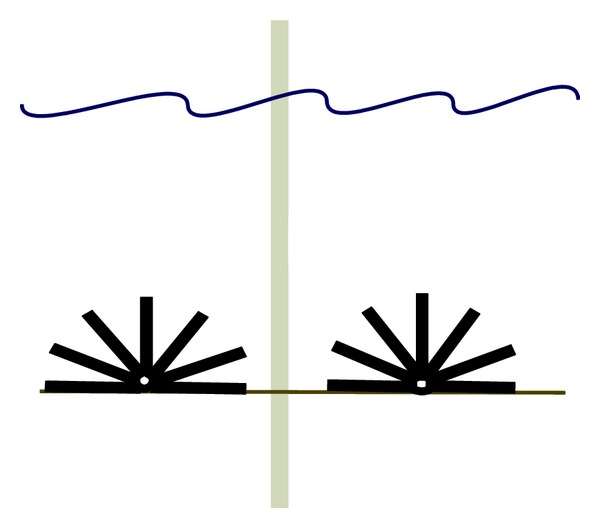
An illustration of the design of the reef system scouring protection around a monopile wind turbine.

There are several designs of scour protection that have been suggested to function both as scour protection and effective artificial reef around wind power foundations. The SeaCult Reef System consists of a circular, perforated concrete cylinder with plastic pipes inserted radially into the perforations (Figure 3) and has a diameter of 6.4 m. The reef may be applied standing, but will be more stable if applied lying down on the seabed in Grip Reef mode. In this mode, parts of the pipes are removed to provide a stable footing. The reefs are used to stimulate marine growth and to control erosion of the seabed. Possible fields of applications are cultivation projects for establishing fishing grounds as artificial habitats and erosion projects in coastal areas and around offshore installations. Field observations during several years (2002–2006) showed high densities of fish and sessile organisms using the structures as habitat, establishing high diverse hard substrate communities [69]. However, low sample size (*n* = 2) just gave an indication and no statistically significant conclusions concerning biodiversity and abundance could be drawn. 

Reef Balls are a specially designed artificial reef used to restore ailing coral reefs and to create new fishing and scuba diving sites [70, 71]. Reef Balls have been applied for beach protection, freshwater, mitigation, and many other uses too. These reefs are about 1.83 m wide and contain around 25–40 holes and a hollow centre. They have been successful within habitat creation both in tropical and temperate waters. The estimated carrying capacity of a reef ball is about 385 kg fish during a year and there will be needed 169 reef balls to cover a scour area [16]. The yearly fish biomass around the base of one wind turbine has been calculated to be 65 000 kg. That shows how efficient that area may become when reef balls are installed compared to control areas. The same case will be expected with the deployment of several SeaCult Reef Systems (Table 1). Even though the expected carrying capacity might be greater than for gravel or boulder protections, negative aspects may be higher expected costs due to specific designs and production processes. 

## 6. Aquaculture and Offshore Energy Installations

The rearing of marine organisms under controlled conditions can be combined locally with offshore energy installations [50, 72]. Thus, it would be within the footprint of the energy installations and should also be applied under environmentally friendly conditions, since sustainability is one of the worries for managers and regulators within the field of renewable energy production. Fishery management is one of the major topics and requires a deeper understanding of ecosystem functioning with its structure and dynamics. An increase in seafood consumption and a decrease in wild fish stocks raise the importance of a higher support for open ocean aquaculture [73]. Today, the most common finfish species used in marine fish farms is salmon [73]. Mussel (*Mytilus* spp.) production is one of the most economically important aspects of global aquaculture and has increased in both the global production and value [74]. In Europe, the annual production of mussels lies about 50% of total the world's production [75]. In Norway kelp has been commercially harvested during the past 40 years by trawling along the coast and the annual harvest is about 170 000 tons. A multifunctional comanagement may be possible for offshore energy installations and open ocean aquaculture where solid turbine foundations can serve as anchor points. In that way both installations will simultaneously use a certain area and may gain both economically, environmentally, and technically. In a review by Buck et al. (2004) a programme for the combination of offshore wind and marine aquaculture has been suggested [72]. The most important points were that (a) a positive spin-off effect may be approached by using existing fishermen with their (modified) boats for the maintenance of wind turbines and for harvesting from aquaculture facilities, (b) local fishermen with their local knowledge have advantages to establish their own aquaculture business, (c) wind foundations can be used to satisfactorily solve anchoring problems by aquaculture structures, and (d) since both constructions share the same area, it is economically efficient to undertake environmental impact assessments.

Furthermore the whole system does not allow fisheries within the offshore farms which are beneficial for the preservation of existing spawning and breeding grounds. Additional hard substrate introduced by the aquaculture structures will be positive for the productivity and diversity of the ecosystem in terms of artificial reef effect. The carrying capacity will be higher and so will density and biomass of several species. 

## 7. Conclusions

When introduced into the marine environment, turbine towers together with their associated scour protection, constitute an artificial reef, and the surfaces are readily colonised by a typical and broadly predictable assemblage of organisms, reflecting zonation patterns observed in adjacent rocky shore communities. Although the scientific literature mostly agreed that there is likely to be a positive effect on fish and crabs, the extent and nature of the effect, it appears, is heavily dependent on the nature of the reef created, the location, and the characteristics of the native populations at the time of introducing the artificial reef. A greater increase in surface area of scour protections by using specially designed material, such as reef ball or SeaCult offshore protections may in fact enhance the reef effect with biodiversity and species richness around the energy devices. Still, a significant research effort is required to predetermine specifically the eventual advantages when creating new habitats and how they can be positive on commercial interesting species such as fish, lobsters, and crabs. To achieve a positive side effect, it is essential that offshore energy installations will be strategically located and monitored carefully. Unfortunately, many artificial reefs have problems to achieve their intentions to enhance biomass production due to lack of knowledge about involved species and habitat requirements of target species. Long-term monitoring of the different scouring protections, gathering data of occurring species and successions, is of high importance. Detailed ecological studies that test the enhancement potential of different types and dimensions of scour protection are necessary. Before developing options for fisheries around closed zones, studies to clarify key questions about target species and the nature of scour protections are needed. An exclusion of trawling in the areas seems to be convenient for both operation of offshore devices and conservation management. Temporarily, fishermen may be affected negatively by no-take zones in and around offshore energy farms. But in a longer term, a spillover of fish and invertebrates to other areas open for commercial fishing is expected. Studies on these spillover organisms using different tagging methods (electromagnetic, acoustic telemetry and conventional) would provide some more exact data on movements, occurrences, and behavioural patterns. Another interesting approach and useful management tool can be to conduct genetic studies for obtaining information about connectivity and spatial population structure of organisms colonising offshore energy devices. 

A multifunctional area that includes mussel farming or seaweed cultivation appears to be one of the most straightforward economic opportunities within existing offshore parks. Still, much research needs to be done to design culture techniques that resist harsh climates in these high energy environments and to combine them with offshore energy installations. 

Habitat loss cannot be mitigated, but compensation can be done either by creating new habitats in the same location or the same habitat somewhere else. There is a good potential for offshore renewable energy installations and their associated scour protection to provide a certain amount of habitat creation. This could have far-reaching benefits for both the local and regional environment, as well as potentially local fisheries using the area. With further work, using more recent data sets and employing modelling techniques, it would also provide a greater argument in the future for the installation of offshore energy installations, strengthening planning applications for future developments.

Finally, different offshore energy parks should cooperate on environmental aspects, based on a BACI design to accelerate application process and to reduce the need to repeat studies. That will help good techniques to reach the market and deliver environmental friendly energy. 

## Figures and Tables

**Table 1:** Net habitat loss and gain through the installation of different types of scour protections around an offshore wind turbine and calculated biomass of expected motile organisms. The expected carrying capacities have been calculated based on reef ball observations. Adapted from [13].

	Habitat loss (m^2^)	Habitat created (m^2^)	Net loss/gain (m^2^)	Biomass per year (kg)
Gravel protection	452	1102	650	19 806
Boulder protection	452	1129	677	20 291
Synthetic fronds	452	439,5	−12,5	7 899
Reef ball	452	3616,6	3164,6	65 000
SeaCult	452	5464	5012	98 203
